# Prevalence of Autism Spectrum Disorder in a large Italian catchment area: a school-based population study within the ASDEU project

**DOI:** 10.1017/S2045796018000483

**Published:** 2018-09-06

**Authors:** A. Narzisi, M. Posada, F. Barbieri, N. Chericoni, D. Ciuffolini, M. Pinzino, R. Romano, M.L. Scattoni, R. Tancredi, S. Calderoni, F. Muratori

**Affiliations:** 1 IRCCS Fondazione Stella Maris, Pisa, Italy; 2 IIER & CIBERER Instituto de Salud Carlos III, Madrid, Spain; 3 Child Neuropsychiatry Unit, ASL 5, Pisa, Italy; 4 Local Education Authority, Firenze, Italy; 5 Research Coordination and Support Service, Istituto Superiore di Sanità, Rome, Italy; 6 University of Pisa, Pisa, Italy

**Keywords:** Autism, child psychiatry, diagnosis, epidemiology, population survey

## Abstract

**Aims:**

This study aims to estimate Autism Spectrum Disorders (ASD) prevalence in school-aged children in the province of Pisa (Italy) using the strategy of the ASD in the European Union (ASDEU) project.

**Methods:**

A multistage approach was used to identify cases in a community sample (*N* = 10 138) of 7–9-year-old children attending elementary schools in Pisa – Italy. First, the number of children with a disability certificate was collected from the Local Health Authority and an ASD diagnosis was verified by the ASDEU team. Second, a Teacher Nomination form (TN) to identify children at risk for ASD was filled in by teachers who joined the study and the Social Communication Questionnaire (SCQ) was filled in by the parents of children identified as positive by the TN; a comprehensive assessment, which included the Autism Diagnostic Observation Schedule-Second Edition, was performed for children with positive TN and SCQ⩾9.

**Results:**

A total of 81 children who had a disability certificate also had ASD (prevalence: 0.79%, i.e. 1/126). Specifically, 66 children (57 males and nine females; 62% with intellectual disability –ID-) were certified with ASD, whereas another 15 (11 males and four females; 80% with ID) were recognised as having ASD among those certified with another neurodevelopmental disorder. Considering the population of 4417 (children belonging to schools which agreed to participate in the TN/SCQ procedure) and using only the number of children certified with ASD, the prevalence (38 in 4417) was 0.86%, i.e. one in 116. As far as this population is concerned, the prevalence rises to 1% if we consider the eight new cases (six males and two females; no subject had ID) identified among children with no pre-existing diagnoses and to 1.15%, i.e., one in 87, if probabilistic estimation is used.

**Conclusions:**

This is the first population-based ASD prevalence study conducted in Italy so far and its results indicate a prevalence of ASD in children aged 7–9 years of about one in 87. This finding may help regional, national and international health planners to improve ASD policies for ASD children and their families in the public healthcare system.

## Introduction

According to the Diagnostic and Statistical Manual of Mental Disorders, fifth edition (DSM-5) (American Psychiatric Association, 2013), Autism Spectrum Disorders (ASD) are a heterogeneous set of neurodevelopmental disorders characterised by deficits in social communication and social interaction plus the presence of restricted interests and repetitive behaviours.

Since the earliest epidemiological investigations in the 1960s, several independent studies have been conducted to determine the prevalence rates of ASD in various geographic regions, with different methodologies that have generated different results (Hill *et al*., 2015). A recent systematic review of worldwide studies found a median prevalence of 0.6% (Elsabbagh *et al*., 2012). In the USA, the Autism and Developmental Disabilities Monitoring Network (ADDM) has performed multiple studies of ASD prevalence rates starting in 2000 (Centers for Disease Control and Prevention, 2007a, 2007b, 2009, 2012, 2014; Christensen *et al*., 2016; Baio *et al*., 2018): the latest estimate of ASD prevalence − one in 59, i.e. 1.7% – represents an increase of 30% compared with the one in 88 rate reported in 2008 and an increase of 50% compared with the one in 150 rate in 2000. The ADDM was conducted in selected US sites and was based on a review of education or healthcare evaluations by trained clinicians. Interestingly, an updated estimate puts ASD prevalence in US children and adolescents at 2.5% in 2014–2016 (Xu *et al*., 2018). This estimate derives from the National Health Interview Survey (NHIS) (Parsons *et al*., 2014), an annual health survey in the USA based on parent report of a physician's diagnosis and conducted on a nationally representative population. However, some substantial differences exist between the ADDM and NHIS investigations –including study design, participant characteristics and methods of data gathering – that make it difficult to compare the two reported prevalence rates directly.

More broadly, the heterogeneity in the reported ASD prevalence may be due to several factors: different methodological approaches (e.g. data source), different measures for assessing ASD symptoms (parent and/or teacher questionnaire alone *v.* direct diagnostic assessment) or different criteria to define an ASD, different study designs for case identification (screening of selected population or retrospective chart review) and different characteristics of the populations studied (e.g., age, ethnicity, availability of services, socioeconomic status). Of note, studies involving more strictly defined autistic symptoms, and/or medical records as the data source and/or a wide age-range found low overall prevalence rates of ASD (Davidovitch *et al*., 2013; Arvidsson *et al*., 2018; Bachmann *et al*., 2018). Conversely, the prevalence of ASD resulted higher in studies including less severe forms of ASD, systematic screening of the general school population and/or prevalence restricted to the child population (Baron-Cohen *et al*., 2009; Kim *et al*., 2011; Romhus *et al*., 2017; Bachmann *et al*., 2018).

Nevertheless, the increase in ASD prevalence over the years is an established and replicated finding (Rice *et al*., 2013). The mechanism underlying the rise of ASD prevalence remains poorly understood and certainly involves multiple factors that can be dichotomised in non-aetiologic (i.e. changes in diagnostic and classification criteria, early diagnosis, service availability, inclusion of milder cases, increased public and scientific awareness and identification) and aetiologic (epigenetic, and environmental risk factors).

For example, as far as non-aetiologic factors are concerned, previous investigations found that changes in diagnostic criteria and inclusion of outpatient data played a relevant role in increased ASD prevalence (Hansen *et al*., 2015; Ramsey *et al*., 2016). Conversely, the contribution of diagnostic substitution of intellectual disability with ASD first proposed by Croen *et al*. (2002) to explain the rise in ASD over recent decades has not been confirmed as a relevant factor in a more recent study (Nevison and Blaxill, 2017).

As regards environmental risk factors, a review of systematic reviews and meta-analyses has been conducted recently (Modabbernia *et al*., 2017). In particular, a positive association between advanced parental age at conception and risk of ASD in offspring was repeatedly found in epidemiological studies (see Wu *et al*., 2017, for a systematic review and meta-analysis on this topic). However, it was estimated that approximately only 5% of the increased prevalence of ASD in a California service system was attributable to older parental age (Shelton *et al*., 2010). In addition, maternal immune activation (MIA) during pregnancy has been detected as a factor that might increase the risk of ASD (Parker-Athill and Tan, 2010), and in particular, MIA caused by asthma and allergies was associated with an increased severity of social symptoms in the offspring (Patel *et al*., 2017). The role of other environmental factors in the increased prevalence of ASD remains more controversial: for example, the majority of epidemiological studies on prenatal exposure to air pollutants, pesticides and phthalates indicated an increased risk for ASD (Hertz-Picciotto *et al*., 2018). However, the different chemical compounds analysed as well as the different exposure metrics used may prevent a direct comparison between studies.

Importantly, further research efforts are needed to better understand and quantify the role played by every single factor in the rise of ASD prevalence as well as their reciprocal influences.

In Europe, the recently reported prevalence of ASD varies considerably between regions and populations. Comparable prevalence rates of ASD were found in Germany (0.4%; Bachmann *et al*., 2018), Poland (0.3% in children aged 0–16 years; Skonieczna-Żydecka *et al*., 2016) and France (0.4% among children aged 7-years old; Van Bakel *et al*., 2015). In these studies, a possible source of ASD prevalence underestimation is that data were derived from registers (Van Bakel *et al*., 2015), from government agencies (Skonieczna-Żydecka *et al*., 2016), or from a German health insurance company (Bachmann *et al*., 2018), without a systematic screening of new cases. As a consequence, individuals with milder autistic symptoms and higher IQ may not have been included. Conversely, higher prevalence rates were detected in other countries, and specifically: 0.6% in Belgian children aged 3–39 months (Dereu *et al*., 2010), 0.7% in Danish children aged 5–10 years (Parner *et al*., 2011), 0.9% in 7–16-year-old children in the Faroe Island (Kočovská *et al*., 2012); 1.1% in 6–11-year-old Irish children (Boilson *et al*., 2016), 1.2% in Icelandic children aged 11–15 years (Saemundsen *et al*., 2013); 1.4% in 0–17-year-old Swedish children (Idring *et al*., 2015).

Crucially, investigations using the same methodology and conducted in different geographical areas are necessary to make a reliable comparison of ASD prevalence between countries. Moreover, repeated investigations that use the same methodology, but are conducted in the same geographical area at different time points are important to produce more precise information about time trends in the prevalence of ASD (Fombonne, 2009).

As far as Italy is concerned, the limited information available on the prevalence rates of ASD is based on registers of previously ASD-diagnosed subjects aged 0–17 years. The percentage of ASD cases ranges from values of 0.05% in the city of Catania – Sicily – (Ferrante *et al*., 2015) to 0.38% in Piedmont (data obtained from the information system NPI.net, 2016) and 0.39% in Emilia-Romagna (data from the information system ELEA, 2016).

The aim of the current study was to estimate the prevalence of ASD in a large representative community sample of the child population in Pisa (Italy). The study protocol we adopted is part of a wider project called ASDEU (Autism Spectrum Disorders in European Union). ASDEU was a 3-year program run by a consortium of 20 centres from 14 countries. ASDEU (http://www.asdeu.eu) has received funds from the Directorate-General of Health and Consumers of the European Commission (DG-SANCO) to increase understanding of and improve responses to autism. One of the main aims of ASDEU was the estimation of autism prevalence in 12 countries in the European Union. In this paper, the Italian prevalence study is reported.

## Method

### Population

The target population was composed of 10 138 children (51.6% males and 48.4% females), living in the metropolitan area of Pisa. Pisa is located in Tuscany (Central Italy) and the metropolitan area has 182 000 inhabitants distributed in the three districts of Pisa, Pontedera and Volterra. The metropolitan area of Pisa has a mean annual income per inhabitant slightly higher than the Italian average (D'onofrio and Murro, 2013). The three geographical areas cover a broad cross-section including urban and rural areas. Each of these districts has a Child Neuropsychiatry Service (CNS); moreover, a national reference center for neurodevelopmental disorders (IRCCS Stella Maris Foundation) is located in the Province of Pisa. The IRCCS Stella Maris Foundation is a tertiary care university hospital specialised in the diagnosis of ASD in early childhood: therefore, we could hypothesise that, for children with suspected/confirmed ASD, the likelihood that parents would look for evaluation or treatment outside the Province of Pisa is minimal.

The population was defined as all children living within the metropolitan area of Pisa whose age, during the period of the study, was between 7 and 9 years (selected by birth year: children born between January 1st 2007 and December 31st 2009), irrespective of gender and ethnicity.

According to Italian law (Law 104/1992), children with any type of disability or emotional and behavioural difficulties generally attend mainstream schools, in ordinary classes at all educational levels, from preschool years until the age of 18. In order to receive a support teacher at the school, parents of children with special educational needs must obtain specific certification, issued by the relevant office, after an extensive assessment has been performed by the local CNS to establish the type of diagnosis and level of disability. Curricular teachers are officially informed about the diagnosis of each child. Certification gives access to (a) a functional diagnosis and clinical profile with an analytical description of the pupil's strengths and weaknesses, and developmental possibilities in the short and medium term; (b) an individualised education plan that includes a description of interventions and activities planned for the pupil in a given period. Classes with one or two children with disabilities contain a maximum of 20 pupils. The inclusion process is supported and periodically verified by a project that defines the objectives and strategies adopted jointly by curricular and support teachers. Support teachers are part of the team of regular class teachers and participate in all regular activities; they are also facilitators of all inclusion processes.

According to the ASDEU protocol and considering the inclusion of all children with disabilities in regular Italian schools, two phases of the prevalence study were performed: (1) detection of the number of certified children with a diagnosis of ASD; (2) identification of new cases.

### Phases of the field study

#### Phase 1. Number of certified children with a diagnosis of ASD

The first step of the study was to obtain information from the Local Unit of the Ministry of Education about the number of 7–9-year-old children with a disability who also had a support teacher according to the Italian Law 104/92. Among these, we identified children with a primary diagnosis of ASD already performed by the local CNS and children with a diagnosis of ASD among those who were certified with other disabilities. This first phase was accomplished by means of a clinical consensus between the ASDEU team and the CNS team; the consensus was reached after verifying the reported diagnosis in clinical records from the CNS. The clinical records included measures of Intelligence Quotient (IQ), language and social skills – obtained through the administration of the appropriate module of the Autism Diagnostic Observation Schedule-Second Edition (ADOS-2; Lord *et al*., 2012).

#### Phase 2. Identification of new cases of ASD by means of a direct school population screening

This second phase was composed of two steps: (a) identification of children at risk for an ASD; (b) clinical assessment of screen-positive children.

### All schools within the metropolitan area of Pisa were invited to participate in the study

In order to increase participation, together with the teacher and parent invitations we included an official letter expressing strong support for the project from the superintendent of schools. Two investigators (A.N. and S.C.) met with teachers and principals to explain the purpose and the importance of the study and encourage their participation.

Two meetings for each district in the metropolitan area of Pisa were organised to describe the study and the instruments. In particular, it was specified that the research team was interested in identifying children with no previous ASD diagnosis. A two-page document describing the study was delivered. The schools that agreed to take part in the study were asked to carry out two tasks: (1) to have teachers fill in the Teacher Nomination form (TN) considering all the 7–9-year-old children in their classes, with the exclusion of certified children; the TN was used as a screening instrument to identify children with no pre-existing ASD diagnosis; (2) to distribute the Social Communication Questionnaire (SCQ) to the parents of children identified by the TN.

#### TN Form

The Teacher Nomination Form (Hepburn *et al*., 2008) contains a list of child characteristics associated with ASD, which were culled from a larger list obtained by brainstorming with clinicians and educators who had extensive experience working with children with autism or with Asperger syndrome. The TN was delivered to teachers who were asked to complete it with regard to the children in their current classes. The TN was estimated to require ~5–10 min per class. Participating teachers were provided with the one-page ‘Teacher Nomination Form’ and asked if any children in their class fit the description, and if so, how many children. Descriptors included the following six items: ‘Socially awkward’; ‘Doesn't seem to understand the feelings of others’; ‘Talks a lot about his/her own interests, but is not very good at conversation’; ‘Doesn't really chat to be friendly’; ‘Not very flexible—Tends to insist on certain rules and routines’; and ‘Is intensely interested in just a few topics or activities’. Then, they were asked to nominate a minimum of two and a maximum of four children in their class who best fit the descriptors on the Nomination Form ranked in order of who fit the description best. The form requested that a minimum of two and a maximum of four children be nominated, regardless of how many children in the class fit the description and even if teachers felt that none of the children really fit the description. The TN strategy was compared with the ASSQ (Autism Spectrum Screening Questionnaire; Ehlers *et al*., 1999) strategy (which takes about 2 h per class) by Hepburn *et al*. (2008), who showed that the proportion of overall agreement between the two measures ranged from 93 to 95% and that the nomination strategy was more sensitive when teachers were asked to nominate at least two children.

#### Social Communication Questionnaire

The SCQ (Rutter *et al*., 2003a) is a 40-item, parent-report screening measure that taps the symptomatology associated with ASD. The items are administered in a yes/no response format and can generally be completed by the parent (or other primary caregiver) in less than 10 min and scored by the administrator in under 5 min. The SCQ Lifetime (used in this study) is completed with reference to the individual's entire developmental history and produces results that are pertinent to referral for a more complete diagnostic assessment. The authors recommend a comprehensive ASD assessment for all subjects who meet or exceed the cutoff score of 15. However, there is no consensus regarding the most effective SCQ score, as it varies according to the characteristics of the study (e.g., age, SCQ version, sample features) (Chesnut *et al*., 2017). Previous studies suggested lowering the SCQ cutoff to 13 (Snow and Lecavalier, 2008), 12 (Corsello *et al*., 2007), 11 (Allen *et al*., 2007), 8 or 7 (Schanding *et al*., 2012), or 7 (Barnard-Brak *et al*., 2016) in order to enhance the accuracy of this instrument. Thus, to reduce the chance of false negatives, for the purposes of this study, we considered children with a total score of 9 or greater as at risk for ASD, in accordance with the ASDEU protocol.

### Clinical assessment of new cases

Step 2.1 consisted in assessing positive cases (TN positive plus SCQ⩾9) in order to confirm or exclude the risk of ASD. The Autism Diagnostic Interview-Revised (ADI-R; Rutter *et al*., 2003b) was used in combination with the ADOS-2. Both instruments were administered by a member of the research team (A.N.), unaware of the exact SCQ score and trained in the use of the ADI-R and ADOS with a high-reliability level. The ADI-R is a semi-structured interview conducted with a primary caregiver of the individual referred for the evaluation of possible ASD. The interview measures behaviours in three areas: social reciprocity, communication and stereotyped behaviours, as well as onset, based on history for children over age 5. The ADI-R includes a scoring algorithm based on the DSM-IV/ICD-10 criteria for autism that yields a classification of autism or non-autism. In order to achieve an ADI-R classification of autism, an individual must meet cut-offs in each area as well as onset criteria.

The ADOS-2 is a semi-structured, standardised assessment of communication, social interaction, play and restricted and repetitive behaviours. It provides a highly accurate picture of current symptoms, unaffected by language. It can be used to evaluate almost anyone suspected of having ASD from 1-year-olds with no speech, to adults who are verbally fluent. For this study, due to the age and IQ level of subjects, we always used Module 3. This Module is intended for subjects with a spontaneous complex speech which is defined as producing a range of sentence types and grammatical forms, using language to provide information about events out of the context of the ADOS and producing some logical connections within sentences. The revised algorithm was used, which consists of two domains, Social Affect and Restricted, Repetitive Behaviours, combined to one score to which thresholds are applied, i.e., >7 for ASD and >9 for autism (Gotham *et al*., 2007). The administration of ADI-R and ADOS-2 was followed by a consensus diagnosis based on information obtained through the ADOS-2 in conjunction with a clinical judgement, according to DSM-5 criteria. Finally, IQ data, measured with the Wechsler Intelligence Scale for Children-Fourth Edition (WISC-IV; Wechsler, 2003) was also collected. The WISC-IV is a scale for assessing cognitive ability, which measures verbal comprehension, perceptual reasoning, working memory and processing speed. After the clinical assessment, only children with a consensus ASD and a total ADOS-2 (Module 3) score ⩾7 were defined as having ASD.

### Ethical permission

The study protocol (version 1–22-11-16) was approved by the Pediatric Ethics Committee of the Tuscany Region in November 2016 (Approval Number: 178/2016).

## Results

### Phase 1. Number of certified children with a diagnosis of ASD

The data provided by the Local Unit of the Ministry of Education informed us that out of 10 138 children (aged 7–9 years) attending schools in Pisa, 239 had a disability certificate. Of these certified children, 66 already had an ASD diagnosis made by the local CNS. Moreover, clinical consensus –based on the analysis of the previous clinical reports and defined as agreement among three clinicians (A.N., F.M., F.B.) – enabled us to identify 15 other children with an ASD diagnosis among those who had a certified diagnosis of other neurodevelopmental disorders (Attention Deficit Hyperactivity Disorder, Intellectual Disability – ID –, Language Disorder, Learning Disorder). This population of 81 (66 plus 15) children of 7–9 years was composed of 68 (84%) males and 13 (16%) females (male-to-female ratio: 5.2:1). Autism severity (evaluated through the calibrated severity scores – CSS –; Gotham *et al*., 2009) was high for 63%, moderate for 16%, and low for 21%. Performance IQ level was >75 for 34%; 41% had a mild ID (>50 IQ <75) and 25% had a severe ID (IQ < 50). As far as expressive language is concerned, 52% of subjects were non-verbal.

Considering that there were 81 children with a confirmed diagnosis of ASD, the ASD prevalence is of 0.8% (95% CI 0.62–0.97) that is 1/126.

### Phase 2. Identification of new cases at risk of ASD by means of direct school population screening

In all, 72 out of 160 (45%) schools joined Phase 2 of the study. The total number of children who took part in Phase 2 was 4417 (43.5% of the 10 138 total number of 7–9-year-old children in the area); of these 4417 children, 111 were certified with disability (Table 1). Thus, in Phase 2, 115 teachers filled the TN Form for 4306 children. Out of these 4306 children, 342 (8%) were nominated through the TN. This result is in line with data from the original investigation of Hepburn *et al*. (2008), in which teachers nominated 8.6% of included children. Parents of these 342 children filled in the SCQ and 49 children (14%; M: 39, F: 10) out of the 342 nominated by teachers obtained a score ⩾9. Children scoring <9 were not sampled for assessment, in accordance with the ASDEU protocol, shared by 12 countries in the European Union.

**Table 1.** Demographic and clinical characteristics of children (*n*  =  81) certified by the local health authority

Gender	% (*n*)
Males	84 (68)
Females	16 (13)
Autism severity^a^	
High	63 (51)
Moderate	16 (13)
Low	21 (17)
Intellectual level	
Average	34 (27)
Mild ID	41 (33)
Severe ID	25 (21)
Expressive language	
Verbal	48 (39)
Nonverbal	52 (42)

ID, intellectual disability.

a According to the ADOS calibrated severity score.

The 49 children at risk (TN positive plus SCQ⩾9) then underwent the clinical assessment procedure. Of these 49 children, ten (20%; M: 8, F: 2) children obtained an SCQ score ⩾15 and six of them agreed to be evaluated: all of these children received a diagnosis of ASD. Seventeen children (35%; M: 12, F: 5) obtained an SCQ score between 11 and 14, and ten of them accepted the evaluation phase, which resulted in a diagnosis of ASD for one child; 22 children (45%; M: 19, F: 3) obtained an SCQ score of 9 or 10, and 14 of them accepted the evaluation phase, in which none of them were diagnosed with ASD. Finally, 30 out of 49 at risk children (60%) were evaluated and seven met algorithm criteria on the ADOS-2 for an ASD diagnosis, confirmed by clinical evaluation. Two subjects did not meet cutoff for autism on the ADI-R. This result is not surprising, since the ADI-R, that is based on DSM-IV criteria (American Psychiatric Association, 2000), provides a dichotomic classification of autism or non-autism, without a cutoff for the broader category of ASD (Rutter *et al*., 2003b). Therefore, the ADI-R may not acknowledge more subtle forms of ASD (Saemundsen *et al*., 2003). Moreover, it is plausible that ADI-R scores are below the cutoff because the parents of these verbal, cognitively-able children were not worried about the neurodevelopment of their child: in fact, children have been referred for a clinical evaluation because of a positive ASD screening, and not because the parents were concerned. It is therefore possible that, in these high-functioning children, ASD symptoms may not be evident at 4.0–5.0 years of age (developmental period investigated in the ADI-R) and become manifest only when social demands exceed patients’ limited capacities (DSM-5; American Psychiatric Association, 2013).

**Fig. 1.**
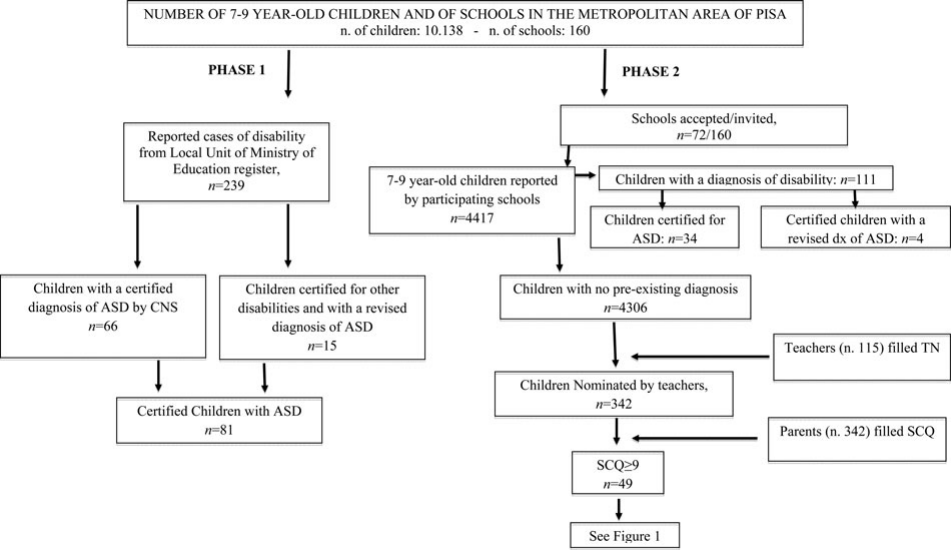
Synopsis of Phase 1 (number of certified children with a diagnosis of ASD) and Phase 2 (number of potential new cases of ASD).

**Fig. 2.**
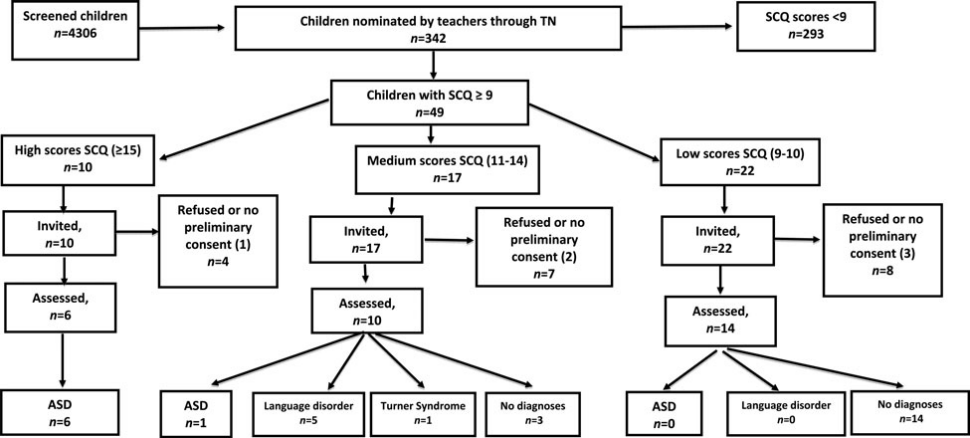
Clinical assessment of children nominated by TN and with a SCQ score ⩾9.

Figures 1 and 2 show the study flow for the screening and evaluation phases.

Probabilistic estimation was used to adjust the prevalence estimates for known non-response to the invitation for assessment within the two score bands where new cases were identified (see Baron-Cohen *et al*., 2009). Using the weightings of 10/6 (ten TN positive children who scored ⩾15 on the SCQ and six children in this score band who participated in the assessment) and of 17/10 (17 TN positive children who scored 11–14 on the SCQ and ten children in this score band who participated in the assessment), the overall directly observed prevalence estimate for ASD was [6 × (10/6)]  +  [1 × (17/10)], which corresponded to 11.7 new (undiagnosed) cases from the screened population.

Moreover, one child living in the geographical target area and attending a school which agreed to participate in the prevalence study arrived for clinical evaluation at the IRCCS Stella Maris Foundation independently from the ASDEU study. She was a 9-year-old girl (negative on the TN) whose parents were worried about her social difficulties, and she received a clinical diagnosis of ASD, which was also confirmed by the ASDEU team. We included this case in the prevalence study as arriving in ‘another way’. Table 2 provides full assessment scores for the eight identified cases of ASD. All had an IQ in the normal range, between 93 and 118.

**Table 2.** Clinical characteristics of the new eight ascertained cases of ASD. For all cases, ADOS-2 Module 3 was used

					ADOS-2				
ID	Age	Gender	SCQ	TN	SA + RRB	SA	RRB	WISC-IV	DSM-5
1	8	Male	12	Nominated	7	5	2	–	ASD
2	8	Male	15	Nominated	8	6	2	93	ASD
3	8	Male	17	Nominated	11	7	4	100	ASD
4	7	Male	28	Nominated	12	9	3	118	ASD
5	8	Male	18	Nominated	15	11	4	112	ASD
6	8	Male	17	Nominated	14	11	3	93	ASD
7	9	Female	16	Nominated	16	12	4	101	ASD
8	9	Female	16	Other way	15	13	2	97	ASD

ADOS-2, Autism Diagnostic Observation Schedule-2; SCQ, Social Communication Questionnaire; TN, Teacher Nomination; SA, Social Affect; RRB, Restricted and Repetitive Behaviours; WISC-IV, Wechsler Intelligence Scale for Children, fourth edition; DSM-5, Diagnostic and Statistical Manual of Mental Disorders, fifth edition.

The prevalence for these new cases with no pre-existing ASD diagnosis ranged from 0.2% (95% CI 0.06–0.33) only considering the evaluated cases, to 0.3% (95% CI 0.12–0.45) considering the probabilistic calculation.

### Summary of the prevalence estimate

Considering the population of 10 138 just using the number of children previously certified as affected by ASD plus the 15 whose diagnosis was modified to ASD by the ASDEU and the CNS teams, the prevalence (81 in 10 138) was 0.8% (95% CI 0.62–0.97) that is 1/126.

Considering the population of 4417 children (belonging to schools which accepted to participate in the TN/SCQ procedure) and using only the number of children certified with ASD (38 in 4417), the prevalence was 0.86% (95% CI 0.59–1.13) that is one in 116. As far as this population is concerned, the prevalence rises to 1% (95% CI 0.74–1.34) if we consider the eight new cases identified in the population of children with no pre-existing diagnoses, and to 1.15% (95% CI 0.83–1.46), that is one in 87, if probabilistic calculation is considered.

## Discussion

Thanks to the first European project (http://asdeu.eu/) designed to estimate ASD prevalence throughout Europe in children aged 7–9 years, our study reveals that one child out of 87 residing within the Province of Pisa (Italy) during 2016, has a DSM-5 diagnosis of ASD. To our knowledge, this is the first epidemiological study in Italy that evaluates the prevalence of ASD in children following a multi-stage methodology. The strengths of this study include the multi-step and multi-informant procedure adopted for ASD identification in a fairly large homogeneous area. Pisa is a province in Tuscany in Central Italy, with a mean annual income per inhabitant slightly higher than the Italian average (D'onofrio and Murro, 2013); in addition, there are no known risk or protective factors for ASD that might lead to variations in prevalence estimates in this province. Moreover, the province of Pisa has a tertiary care university hospital with a Unit specifically dedicated to the diagnosis and treatment of ASD children; therefore, we could assume that the propensity to seek health care for young subjects with ASD outside of the province of Pisa is minimal.

We used a multi-stage design for case identification, which consisted in: (a) consultation of existing records in education service provider databases for certified ASD diagnoses in children aged 7–9 years; (b) analysis of these records by means of a consensus between the ASDEU team and the child neuropsychiatry team responsible for the case, in order to verify the appropriateness of the neurodevelopmental diagnosis inserted into the database; (c) administration of two sequential screening tools (TN filled in by teachers and SCQ filled in by parents) with the aim of identifying the risk for ASD among 7–9-year-old undiagnosed children; (d) in-depth clinical evaluation with standard diagnostic instruments, such as the ADOS-2, of at-risk children by professionals with over 15 years experience in assessing and diagnosing ASD. This methodology gave us a fairly complete picture of the distribution of autistic disorders within the spectrum.

The first two steps (that is phase 1 of the study) provided epidemiologic data regarding those ASD children who require help during their everyday life. In fact, all these children were certified as needing a support teacher, even if they showed good cognitive skills. The last two steps (that is phase 2 of the study: screening a typical school population) also enabled us to detect individuals with ASD who were not in contact with health services, and thus to increase the identification of ASD cases.

Using a similar procedure, Kim *et al*. (2011) screened the entire 7–12-year-old population of a South Korean community (*n*  =  36 592 children attending elementary schools plus *n*  =  294 children enrolled in the disability registry and special education schools) by administering the Autism Spectrum Screening Questionnaire (Ehlers *et al*., 1999) and a comprehensive ASD assessment battery (i.e. ADOS, ADI-R, cognitive tests) to screen-positive children. The authors found an ASD prevalence of 0.7% in the high-probability group (i.e. special education schools/disability registry), comparable with our group of certified children, and a large number of unidentified cases attending regular schools, with an ASD prevalence of 1.9% in this population.

Our results are consistent with those identified by Kim *et al*. (2011) as regards the prevalence estimate of the certified group (0.8% in our study *v.* 0.7% children enrolled in special education schools/disability registry in Kim's study). Also some characteristics of our sample were similar to those reported by Kim *et al*. (2011): for example, our male-to-female ratio is 5.2:1 *v.* the 5.1:1 found by Kim *et al*. (2011) and the percentage of children with severe intellectual disability was only slightly superior in our group than in Kim's study (25 *v.* 19%). This prevalence of certified children is of particular importance, considering that Italy is one of the first European countries promoting school inclusion for ASD.

In contrast, significant differences emerge as far as ASD prevalence in the general population is concerned, in that the prevalence in our study (0.3%) is fairly low compared with the one in Kim's study (1.9%). A number of reasons could underlie these differences. First, we used a very conservative approach to diagnosis. In fact, only children with an ADOS-2 score above the cutoff for ASD and a DSM-5 diagnosis of ASD were considered as having ASD. Moreover, we used the DSM-5 criteria, which are more restrictive than those of the DSM-IV used by Kim *et al*. (2011), who included also children with the controversial diagnosis of Pervasive Developmental Disorders Not Otherwise Specified. Indeed, 74% of cases in the general population sample were in the ‘other ASD’ category and not in the ‘Autistic Disorder’ category (Kim *et al*., 2011). The same problem occurs for the prevalence study by Baron-Cohen *et al*. (2009), which includes in the autism spectrum condition children with an ADOS score under the cutoff for the spectrum. Conversely, we only considered children with autism spectrum disorders, not conditions, and with an ADOS-2 score above the cutoff for ASD.

Nevertheless, we have to consider that we may have missed cases among screen-negative children on the TN or SCQ. This limit is reduced as far as TN is concerned by the demonstrated agreement (Hepburn *et al*., 2008) between the TN and the ASSQ, which has been used in many other studies, and as far as SCQ is concerned, by the fact that we considered the lower cutoff point of 9 instead of the official cutoff of 15. In particular, we cannot exclude an underestimation of females with autism. In fact, while in the study by Kim *et al*. (2011) the male-to-female ratio was 2.5:1, in our general population study it is 3.3:1. The fact that using our screening tools (TN and SCQ) only one female child received an ASD diagnosis, after clinical assessment, is in line with the gender ratio (8:1) of high-functioning ASD individuals (Hill *et al*., 2015), but it can also be an index of under-identification of females with ASD during screening procedures. Indeed, a further female child received an ASD diagnosis, but she screened negative on the TN. This poses the question of false negative female children who are on the spectrum. In regard to this issue, the literature indicates a gender bias in ASD diagnosis: in fact, girls with a normal intellectual level, who are not impaired by behavioural problems are less likely to receive an ASD diagnosis than boys, even at comparable levels of autistic symptoms (Dworzynski *et al*., 2012). In fact, females with ASD may have more socially accepted interests (Van Wijngaarden-Cremers *et al*., 2014), and may be more likely to have developed coping strategies to manage social situations (Dean *et al*., 2016). Moreover, a larger proportion of verbally fluent females with ASD, compared with their male counterparts, receive scores in the non-ASD range on the ADOS-2 Module 3 algorithm, indicating that some females with ASD may be missed also by this instrument (Bishop *et al*., 2017).

Our findings have to be interpreted in the context of other limitations. First, we used first-level screening instruments (TN and SCQ) to determine which children needed further evaluation to determine an ASD diagnosis. The administration of two sequential screening tools (TN and SCQ) aimed to increase the specificity (i.e. to reduce false positives): in fact, this sequential procedure involved applying a second screening test (SCQ) to children who initially screened positive (TN). In this way, an erroneous positive identification with consequent family stress as well as time-consuming assessment procedures is avoided (Charman and Gotham, 2013). Nevertheless, an underestimation of ASD, particularly in high-functioning subjects who do not give teachers and/or parents cause for concern cannot be excluded. In fact, teachers and parents may be focused mainly on academic achievements, and fail to recognise or minimise the socio-communicative difficulties as well as the peculiar and restrictive interests of these children. In accordance with this view, recent literature suggests there may be a delay in obtaining an ASD diagnosis for children with average IQ scores (Jónsdóttir *et al*., 2011; Mazurek *et al*., 2014; Romhus *et al*., 2017). These individuals might receive a first ASD diagnosis in adolescence or adulthood, or may be referred to mental health services, not for core ASD symptoms, but for comorbid psychiatric disorders (Aggarwal and Angus, 2015), which are frequent in high-functioning adolescents (Strang *et al*., 2012) and adults (Lugnegård *et al*., 2011) with ASD. Other high-functioning subjects could be incorrectly diagnosed (Lugnegård *et al*., 2012; Takara *et al*., 2015) or never be diagnosed with an ASD (Brugha *et al*., 2011) and this evidence leads respectively to inappropriate treatment or missed opportunities to treat the disorder.

Second, another possible source of ASD prevalence underestimation is the absence of parental consent for further evaluation of all the children screened positive on the TN and SCQ. Therefore, some children, despite being at risk of ASD, did not receive a clinical assessment for suspected ASD. One possible explanation for parental refusal of a direct clinical evaluation requiring both parent and child attendance is the presence of logistical difficulties (e.g. taking time off work, transportation, childcare for sibling/s). Another possibility is that, despite increasing public awareness and decreasing stigmatisation surrounding ASD in recent years (Kapp *et al*., 2013; DeVilbiss and Lee, 2014), some parents could be reluctant to accept an in-depth neuropsychiatric evaluation for their intelligent child, and therefore they prefer to refuse the diagnostic assessment.

Third, an additional cause of underdiagnosis could derive from the absence of further evaluation among children who screen negative on the TN or SCQ. Indeed, the TN, despite being a time-efficient and cost-effective way to screen children in schools (Hepburn *et al*., 2008), is certainly less able to detect ASD than more universal screening procedures, which can identify subjects who have symptoms that are subtle, subthreshold or attributed to other conditions (Schanding *et al*., 2012). Analogously, the SCQ is an acceptable screening instrument for ASD, and its accuracy is enhanced when the Lifetime version is used in a population older than 4 years of age (Chesnut *et al*., 2017), as in our sample. However, even if we chose a relatively inclusive SCQ cut-off score (i.e., able to identify the majority of individuals with ASD, but also including subjects who are not), we may still have missed cases among children with an SCQ score <9. Indeed, Barnard-Brak *et al*. (2016) calculated that using a cutoff of 7 on the SCQ, 5.8% of children were incorrectly identified as not having ASD (e.g., false negatives). For this reason, some authors have suggested lowering the cutoff score to 8 or 7 in order to detect high-functioning subjects (Schanding *et al*., 2012), or to a score of 7 for school-aged elementary individuals (Barnard-Brak *et al*., 2016).

Overall, the aspects above-mentioned may have contributed to the lower ASD prevalence obtained in comparison with other studies conducted in other countries. However, one of the strengths of our study is that it provides the most up-to-date epidemiological investigation of ASD in Italy and that it is one of the first investigations using the strict DSM-5 criteria for ASD (Morales-Hidalgo *et al*., 2018). Another strength is related to the procedure that is different from other prevalence studies in which ASD cases have been identified based on existing health records (Taylor *et al*., 2013), service provider databases (Croen *et al*., 2002), or on parent report alone (Blumberg, *et al*., 2013; Zablotsky *et al*., 2015), without any additional direct diagnostic clinical evaluation. Even compared with the studies that used a similar diagnostic methodology to ours (e.g., Baron-Cohen *et al*., 2009), the final criteria we used for assigning an ASD diagnosis to children found positive on the TN and SCQ were more stringent as all subjects had to meet ASD cutoffs on the ADOS-2 Module 3 algorithm, in addition to the DSM-5 diagnostic criteria for ASD. In the study by Baron-Cohen *et al*. (2009) instead, not only disorders but also conditions were considered.

In conclusion, this study indicates a prevalence of ASD in 7- to 9-year-old children living in a defined large catchment area in Italy, of about one in 87. This prevalence covers a wide spectrum of autistic disorders using DSM-5 criteria, from children with a clear diagnosis (who in Italy attend regular schools with a support teacher) to children with no pre-existing ASD diagnoses. This result indicates the need to implement, in line with the estimated prevalence, specialised services and multidisciplinary ASD teams (within the public healthcare system) able to address the varied needs (in term of diagnosis, rehabilitative intervention and care) of the different autisms within the spectrum.

## Availability of data and materials

The data collected in the present study contain sensitive information on a very vulnerable group, i.e., children with ASD. Even though the data have been anonymised, they still contain enough details to enable identification of single individuals. Therefore, in order to approve the study, the Regional Ethical Review Board in Florence (Tuscany) made considerable restrictions regarding data access. This means we will not be able to share our data with other researchers outside the ASDEU group.
